# *Tamarixia radiata* Behaviour Is Influenced by Volatiles from Both Plants and *Diaphorina citri* Nymphs

**DOI:** 10.3390/insects10050141

**Published:** 2019-05-16

**Authors:** Yan-Mei Liu, Shu-Hao Guo, Fei-Feng Wang, Li-He Zhang, Chang-Fei Guo, Andrew G. S. Cuthbertson, Bao-Li Qiu, Wen Sang

**Affiliations:** 1 Key Laboratory of Bio-Pesticide Innovation and Application, Department of Entomology, South China Agricultural University, Guangzhou 510640, China; yml759473267@163.com (Y.-M.L.); guoshuhaozd@163.com (S.-H.G.); wff1013634299@163.com (F.-F.W.); baileyqiu@scau.edu.cn (B.-L.Q.); 2 Engineering Research Center of Biological Control, Ministry of Education, Guangzhou 510640, China; shengfang447@126.com (L.-H.Z.); changfeiguo@163.com (C.-F.G.); 3 Independent Science Advisor, York YO41 1LZ, UK; andrew_cuthbertson@live.co.uk

**Keywords:** *Tamarixia radiata*, asian citrus psyllid, olfactory, nymphal instar, *Murraya paniculata*

## Abstract

*Tamarixia radiata* (Waterston) is an important ectoparasitoid of the Asian citrus psyllid, *Diaphorina citri* Kuwayama, a globally destructive pest of citrus. In the present study, a Y-tube olfactometer was employed to investigate whether the parasitoid *T. radiata* is capable of utilizing the odour source emitted by both plants and insect hosts during its foraging. The odour sources included *Murraya paniculata* (L.) shoots, 1st, 2nd, 3rd, 4th, and 5th *D. citri* instar nymphs, both individually and in combinations. Moreover, nymph-stage choice for parasitism, including 3rd, 4th, and 5th *D. citri* instar nymphs, was carried out. The results indicated that female *T. radiata* were only significantly attracted to volatiles emitted by *M. paniculata* shoots, 3rd, 4th, and 5th instar nymphs of *D. citri*, but could not distinguish between them. *T. radiata* males were not attracted by odours sourced from any instar *D. citri* nymphs. Female *T. radiata* adults exhibited a significant preference to later instar nymphal stages of *D. citri* for oviposition. The results from this study can be used to guide further investigations on the searching behaviour of this parasitoid and its utilization in *D. citri* biocontrol.

## 1. Introduction

Huanglongbing (HLB) is one of the most destructive diseases of citrus fruit, to the extent that it is a threat to the global industry [1]. The Asian citrus psyllid, *Diaphorina citri* Kuwayama (Hemiptera: Psyllidae), is a destructive pest of citrus, because of its ability to transmit *Candidatus* Liberibacter asiaticus (CLas), the causal agent of HLB disease [1,2,3,4]. All commercial citrus cultivars are vulnerable to HLB. There is currently no cure for HLB [4]. The epidemic of HLB relies on the movement and density of *D. citri* [5]. Both *D. citri* adults and nymphs can acquire CLas after feeding on infected plants for a period of time, but nymphs are much more efficient at this [6]. The management of HLB is presently reliant on the control of *D. citri* and on the use of healthy trees, as well as the elimination of HLB-symptomatic plants [7].

*Tamarixia radiata* (Waterston) (Hymenoptera: Eulophidae), an ectoparasitoid, is an effective parasitoid of the Asian citrus psyllid [8]. A single female *T. radiata* adult can deposit up to 19 eggs per day and a total of 300 eggs during her lifetime [9,10,11]. It has been reported that a single female *T. radiata* may kill up to 500 *D. citri* nymphs during her lifetime by parasitism and direct predation of the adults [12]. This parasitoid has been successfully introduced into La Reunion Island and Guadeloupe Islands, where it has considerably reduced populations of *D. citri* [13,14]. As a result, it is considered one of the most important biological control agents of *D. citri*.

The interactions between hosts and parasitoid insects in a microhabitat are very complex. Therefore, successful host discrimination plays an important role in the process of a female parasitoid parasitizing [15]. The foraging behavior of a female parasitoid often undergoes several steps: (1) habitat search, (2) host community location, (3) host location, (4) host examination, and (5) host acceptance [16]. Parasitoids generally use multiple sensory modes to distinguish their hosts, such as visual, olfactory, touch, and other elements. Among these, olfactory mode is a well-studied mechanism as a great number of parasitoids utilize chemicals (volatiles) to forage for their host [17,18]. For example, female *Fopius arisanus* (Sonan), an egg-pupal parasitoid of fruit flies, has been shown to be attracted to mango leaves in olfactory location tests [19]. *Polistes sulcifer* (Zimmermann) was able to perceive nest odour and discriminate different species of *Polisters dominulus* (Christ) [20].

To date, several studies have been conducted on volatiles used by *T. radiata* [21,22,23,24]. Mann et al. [8] investigated the behavioral response of *T. radiata* to olfactory stimuli emitting from mixed 4th and 5th instar nymphs, and adults as well as citrus. They found that female *T. radiata* responded positively to the odours emitted from mixed 4th and 5th instar nymphs of *D. citri*, but did not respond to odours emitted from *D. citri* adults or citrus [8]. These results indicated that female *T. radiata* may use volatiles emitted from mixed 4th and 5th instar nymphs of *D. citri* to locate their host. However, information of *T. radiata* responses to other host plants and different instar nymphs of *D. citri* is lacking. Therefore, in the present study, we investigated the behavioral response of *T. radiata* to olfactory stimuli volatilizing from *Murraya paniculata* (L.) plants and different instar nymphs of *D. citri*, respectively. We also undertook a pair comparison olfactory choice using different developmental nymphal stages as odours to investigate whether wasps can distinguish nymphs from different developmental stages. Finally, we conducted a host preference parasitism test by using the different developmental nymphal stages to investigate whether there exists a relationship between olfactory and host insect preference for parasitism of *T. radiata*.

## 2. Materials and Methods

### 2.1. Host Plants

*Murraya paniculata* (L.) seedlings were planted in plastic pots (30 × 30 cm) in a glasshouse under ambient conditions (20–35 °C, r.h. 56%–85%) at the campus of South China Agricultural University (SCAU), Guangzhou, P. R. China. The seedlings were irrigated and fertilized regularly as required.

### 2.2. Insect Rearing

*Diaphorina citri* Kuwayama colonies were collected from a natural infection on *M. paniculata* plants in SCAU in April 2017 and reared on *M. paniculata* under laboratory conditions (26 ± 1 °C, r.h. 80% ± 10% with L/D = 14:10 photoperiod). *M. paniculata* flushes were used to stimulate oviposition of *D. citri*. In brief, 15 flushing *M. paniculata* plants were moved into a cage (60 × 60 × 60 cm). Then, 4000 *D. citri* were released and maintained inside the cage for 72 h to allow for spawning. Subsequently, the plants with *D. citri* eggs were placed into a clean cage for egg hatching and nymphal development. Nested-quantitative Polymerase Chain Reaction (PCR) detection method, as described by Coy et al. [25], was used to confirm *D. citri* CLas-free.

*Tamarixia radiata* (Wollaston) colonies were initiated from *D. citri* hosts on *M. paniculata* in SCAU in May 2017. This colony of *T. radiata* was reared on a *D. citri*–*M. paniculata* system in a glasshouse (26 ± 1 °C, r.h. 80% ± 10% with L/D = 14:10 photoperiod). Flush shoots with mummified nymphs of *D. citri* were cut and placed into petri dishes (12 cm) for rearing. After *T. radiata* adults emerged, they were reared on a glass tube under laboratory conditions and provided with a 20% sugar solution. Therefore, *T. radiata* adults conducted their mating behavior either in the dishes or the tubes, as Chen and Stansly [26] reported that *T. radiata* finish their mating behavior during the first two days following emergence. Prior to bioassays, the mated adult wasps were segregated into 1 mL glass tubes and provided with 20% sugar solution.

### 2.3. Olfactometer Behavioral Assay

Responses of *T. radiata* to different plant and *D. citri* nymphal volatiles were determined in a Y-tube olfactometer. The olfactometer contained a Y-shaped glass tube with a 0.8 cm inner diameter. Both the base and the two arms of the Y-tube were 6 cm in length. The airflow through each of the two arms of the Y-tube was kept constant at 80 mL min^−1^. A vacuum pressure pump pushed room air through activated charcoal and a 500 mL Pyrex Erlenmeyer flask containing distilled water. Assays were conducted in a room at 26 ± 1 °C and r.h. 60% ± 10%. The light intensity of the Y-tube under a red light in the experimental environment was approximately 12 lux.

*T. radiata* adults within 5–9 d old were introduced individually at the base of the Y-tube. They were observed for 300 s. When parasitoids entered the 1/3 arms of the olfactometer and successively remained for at least 1 min, it was recorded as a choice between the odour treatments. The odour source (different stages of *D. citri* nymphs or *M. paniculata* shoots with no water) was disposed into a glass bottle. The different life stages of *D. citri* nymphs or *M. paniculata* shoots with no *D. citri* nymphs were tested separately. Then, an odour source was randomly appointed to one arm of the olfactometer at the beginning of each bioassay and was reversed after every 10 wasps to remove positional bias. At least forty *T. radiata* were run before changing the odour source. Wasps were also exposed to clean air versus clean air in the olfactometer in order to verify the absence of positional bias before odour testing; the results indicated that there was insignificant choice in the clear air versus clear air test. After treatments, all equipment along with the Y-tube was thoroughly cleaned with soap solution and deionized water and finally baked in a dryer at 75 °C for 12 h. A minimum of 40 *T. radiata* adults was examined per treatment combination. Female and male *T. radiata* were tested separately.

### 2.4. Host Foraging Behaviour of T. radiata to Volatiles from M. paniculata Shoots and D. citri Nymphs

The following odour source groups were conducted: (1) the 1st instar nymphs of *D. citri* versus clean air; (2) the 2nd instar nymphs of *D. citri* versus clean air; (3) the 3rd instar nymphs of *D. citri* versus clean air; (4) the 4th instar nymphs of *D. citri* versus clean air; (5) the 5th instar nymphs of *D. citri* versus clean air; (6) *M. paniculata* shoots versus clean air; (7) 5th instar nymphs of *D citri* versus the 4th instar nymphs of *D. citri*; (8) 5th instar nymphs of *D. citri* versus the 3rd instar nymphs of *D. citri*; (9) the 4th instar nymphs of *D. citri* versus the 3rd instar nymphs of *D. citri*. A total of 500 1st instar, 2nd instar, 3rd instar, 4th instar, and 5th instar nymphs, respectively, of *D. citri* were used in the different *D. citri* nymphal olfactometer assay comparisons. In addition, 17 g of *M. paniculata* shoots with no *D. citri* were used in the *M. paniculata* olfactometer assays.

### 2.5. Asian Citrus Psyllid Host Parasitizing Preference of T. radiata

The preference of *D. citri* nymphs by female *T. radiata* was determined by conducting a parasitizing choice test between 3rd, 4th, and 5th nymphal stages. The groups of the parasitizing choice test were as follows: (1) 3rd versus 4th instar nymphs; (2) 3rd versus 5th instar nymphs; (3) 4th versus 5th instar nymphs; and (4) 3rd versus 4th versus 5th instars nymphs. Fifteen randomly selected nymphs were transferred onto the shoots of *M. paniculata* using a camel hair brush in a nylon bag (80 mesh/cm^2^). One 5 d old mated female *T. radiata* was introduced to the nylon bag for parasitizing over a 24 h period before being removed. *Murraya paniculata* plants with *D. citri* and *T. radiata* were then moved to the laboratory and maintained at 26 ± 1 °C, r.h. 80% ± 10% with L/D = 14:10 photoperiod. The number of eggs laid by *T. radiata* was identified and counted immediately using a stereomicroscope (Zeiss, Shanghai, China). In this experiment, there were six replicates (*T. radiata* parasitoid) for each instar nymphal treatment.

### 2.6. Data Analysis

Datasets were analyzed using analysis of variance via associated computer software (SPSS 17.0). For the Y-tube olfactometer assays, χ^2^ tests were used to analyze the datasets collected from the olfactometer behavioral assays. Student’s *t*-test was applied to analyze the significant difference of data obtained from nymph-stage preference assays, including the following: (1) 5th versus 4th instar nymphs; (2) 5th versus 3rd instar nymphs; and (3) 3rd versus 4th instar nymphs. In addition, the numbers of hosts parasitized in 3rd versus 4th versus 5th instar nymphs were compared using the Least Significant Difference (LSD) multiple range test (*p* < 0.05).

## 3. Results

### 3.1. Host Foraging Behaviour of T. radiata to Volatiles from M. Paniculata Shoots and D. citri Nymphs

**Figure 1:**
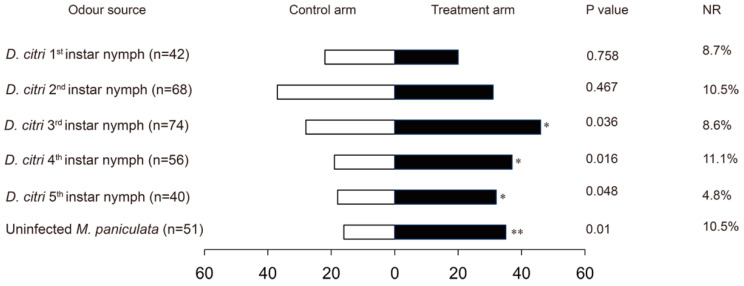
Response of female *Tamarixia radiata* to odours emitted from *Murraya paniculata* or *Diaphorina citri* nymphs versus blank controls, respectively, within a Y-tube olfactometer. * indicates significant differences between the white bars and black bars (* *P* < 0.05, ** *P* < 0.01). NR: percentage of non-responders.

**Figure 2:**
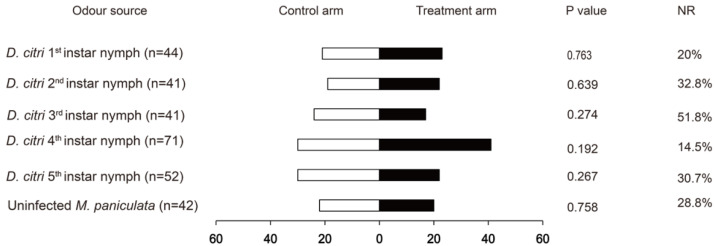
Response of male *Tamarixia radiata* to odours emitted from *Diaphorina citri* nymphs versus blank controls with a Y-tube olfactometer. NR: percentage of non-responders.

The choices of *T. radiata* for host plants and insects are shown in Figure 1 and Figure 2. The results demonstrate that *T. radiata* female adults exhibited a significant response to the *M. paniculata* shoots (χ^2^ = 7.078, df = 1, *P* = 0.01), as well as 3rd instar (χ^2^ = 4.378, df = 1, *P* = 0.036), 4th instar (χ^2^ = 5.786, df = 1, *P* = 0.016), and 5th instar (χ^2^ = 3.92, df = 1, *P* = 0.048) nymphs of *D. citri* compared with the blank odour control (Figure 1). In comparison, the female wasps did not prefer either the 1st instar (χ^2^ = 0.095, df = 1, *P* = 0.758) or 2nd instar (χ^2^ = 0.529, df = 1, *P* = 0.467) nymphs of *D. citri* odour treatments. In contrast, the male wasps were not attracted to odours from any of the nymphal stages of *D. citri* and *M. paniculata* shoots (1st instar: χ2 = 0.091, df = 1, *P* = 0.763; 2nd instar: χ2 = 0.220, df = 1, *P* = 0.639; 3rd instar: χ2 = 1.195, df = 1, *P* = 0.274; 4th instar: χ2 = 1.704, df = 1, *P* = 0.192; 5th instar: χ2 = 1.231, df = 1, *P* = 0.267; *M. paniculata* shoots: χ2 = 0.095, df = 1, *P* = 0.758) (Figure 2).

**Figure 3:**
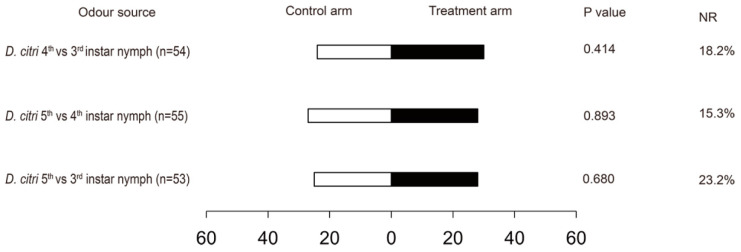
Response of female *Tamarixia radiata* to odours emitted from *Diaphorina citri* 5th vs. 3rd, 5th vs. 4th, and 4th vs. 3rd instar nymphs with a Y-tube olfactometer. NR: percentage of non-responders.

The female wasps were only attracted by the 3rd, 4th, and 5th instar nymphs, but whether the wasps could distinguish between these nymphal instars was not clear. Therefore, pair comparison tests were performed. Here, the different developmental nymphal stages were used as odours. The results showed that there were no significant differences in attraction by the comparison of 3rd and 4th instar nymphs (χ2 = 0.667, df = 1, *P* = 0.414), 3rd and 5th instars nymphs (χ2 = 0.170, df = 1, *P* = 0.680), and 4th and 5th instar nymphs (χ2 = 0.018, df = 1, *P* = 0.893) (Figure 3).

### 3.2. Asian Citrus Psyllid Host Parasitizing Preference of T. radiata

The parasitism preference of *T. radiata* for different nymphal stages of *D. citri* is shown in Table 1. The results demonstrate that *T. radiata* can oviposit on all tested nymphal stages of *D. citri*. However, there was a significantly higher parasitization rate of 5th instar nymphs compared with that of 3rd instar nymphs (F = 4.827, df = 8, *P* = 0.025). Furthermore, there was a significantly higher parasitization rate of 5th instars nymphs compared with that of 4th instar nymphs (F = 0.263, df = 4, *P* = 0.004). When 3rd and 4th instar nymphs were analyzed, it was shown that higher numbers of 4th instar nymphs were parasitized by the wasps (F = 2.290, df = 8, *P* <0.001). When the 3rd, 4th, and 5th instar nymph were analyzed together, there was a significantly higher parasitization rate of 5th instars nymphs of *D. citri* (F = 15.496, df = 2, 11; *P* = 0.001) (Table 1).

## 4. Discussion

Location and choice of hosts is an elementary part of the life of parasitoids. This determines the population success of their offspring. Generally, the location and choice of an oviposition site are mainly related to visual, olfactory, and tactile responses [19,27,28]. In this study, we investigated the olfactory response of *T. radiata* on all instar nymphs of *D. citri* and *M. paniculata* shoots.

Male *T. radiata* did not respond positively to different nymphal life-stage olfactory cues. In regards to female *T. radiata*, volatile cues emitted by 3rd, 4th, and 5th instar nymphs and *M. paniculata* shoots proved attractive within the olfactory tests. However, the female *T. radiata* could not distinguish between 3rd, 4th, and 5th instars nymphs, although our study showed them to have a preference to parasitize 5th or 4th instars nymphs compared with 3rd instar nymphs.

Attraction chemicals can be divided into two groups; that is, either long-range or short-range orientation. Parasitoids use synomones such as volatiles from the plant directly or indirectly via herbivore-induced mechanisms to locate their habitat (long-range searching). Volatiles that are emitted from the host insect or its by-products are used as kairomones in the process of host location (short-range searching) [17,28,29]. Chemicals produced by plants may influence the searching behavior of parasitoids. Specifically, plant-produced chemicals play a more important role in long-distance searching [30,31,32]. Therefore, some parasitoids utilize volatiles produced by undamaged plants to locate the habitat of their host, which may benefit both plants and parasitoids for normal survival and development [33,34,35]. The present results from the Y-tube olfactometer assays distinctly show that female *T. radiata* can positively respond to chemical cues emanated by *M. paniculata* shoots and their host nymphs. Such attraction indicates a high searching efficacy of foraging female *T. radiata* within the given habitat. These results suggest that female *T. radiata* may effectively respond to synomones from *M. paniculata* trees in order to locate suitable host habitat (long-range searching). Following this, female *T. radiata* may then refine their search by utilizing kairomones emanated by *D. citri* larvae nymphs from the host habitat (short-range searching). Studies conducted with *Cotesia urabae* Austin & Allen have also found that parasitoid wasps are capable of responding to volatiles emanated by plants of their hosts and the insect hosts themselves [36]. Specifically, Avila et al. [36] observed that female *C. urabae* showed attractions to odour sources emitted by *Eucalyptus fastigata* Deane & Maiden leaves, their host larvae (*Uraba lugens* Walker), the target plant–host complex, and *E. fastigata* leaves from feeding damage caused by their host larvae [36].

In the current study, the behaviour of female *T. radiata* was significantly attracted by volatiles emitted from *M. paniculata*. This result is different from Mann et al.’s results, in which the response of *T. radiata* females was not significantly attractive to intact *M. paniculata* that weighed ~2 g [8]. This would suggest that it is very concentration-sensitive. In our study, the female *T. radiata* responded significantly to the volatiles from a group of ~17 g mass *M. paniculata* wattle. It appears, therefore, that the volatiles from an ~2 g mass are not sufficient to lead to a significant attraction in the movement activity of female *T. radiata.* Thus, this parasitoid probably has a responsive concentration threshold. Similar studies conducted with *Trichogramma brassicae* Bezdenko have also found that there is a sensitive concentration threshold for *T. brassicae* to respond to kairomones from egg and female adult stages of *Ostrinia nubilalis* Hubner. More specifically, the parasitoids responded to the volatiles from batches of three egg masses but did not respond to a single egg mass [37]. Alternatively, *M. paniculata* shoots possibly may induce volatiles following mechanical damage because the tested *M. paniculata* shoots were cut from *M. paniculata* trees. For this reason, the female *T. radiata* were probably attracted by volatiles induced by mechanical wounding. Similar results have been reported by Cruz-Lopez et al. [33], who found that the amount of linalool and methyl salicylate increased in mechanical damage of Robusta coffee berries. Furthermore, two parasitoids (*Prorops nasuta* Waterstone and *Phymastichus coffea* LaSalle) were attracted to the methyl salicylate and linalool [33].

Volatiles emitted from 3rd, 4th, and 5th instar larval nymphs of *D. citri* caused a significant activation of parasitoid choice, but volatiles emitted from 1st and 2nd instar nymphs did not exhibit a significant attraction to female *T. radiata*. It would appear that *T. radiata* can be stimulated to move upwind toward the odour source emitted from 3rd, 4th, and 5th instar larvae nymphs. This phenomenon may be explained by the oviposition behavior of *T. radiata* and the different mass of different instar nymphs of *D. citri*. Alternatively, Li et al. [11] reported that the average daily fecundity of *T. radiata* in 3rd, 4th, and 5th instar nymphs was at least 8.2 eggs/female, whereas in 1st and 2nd instar nymphs, parasitism was not recorded. With regard to the larvae stages of the hosts, a comparison of the olfactory choice indicates that larval nymphs of *D. citri* liberate a kairomone. In addition, considering all the host–parasitoid couples investigated, kairomones may be produced by 3rd, 4th, and 5th instar larval nymphs of *D. citri.* Therefore, there exists a mixture of one or more molecules that respond positively to activate the activity of female *T. radiata.* Alternatively, the phenomenon in which female *T. radiata* were not significantly attracted by volatiles emitted from 1st and 2nd instar nymphs may also be associated with the lower mass of 1st and 2nd instar nymphs compared with that of 3rd, 4th, and 5th instar nymphs. It appears, therefore, that the volatiles from a lower mass are not sufficient to lead to a significant attraction in the movement activity of female *T. radiata.* Thus, this parasitoid probably has a responsive concentration threshold. Similar studies conducted with *T. brassicae* have also found that there is a sensitive concentration threshold for *T. brassicae* in responding to kairomones from egg and female adult stages of *Ostrinia nubilalis*. More specifically, the parasitoids responded to the volatiles from batches of three egg masses but did not respond to a single egg mass [37]. Finally, Chen and Stansly [26] found that both female and male *T. radiata* could feed on the hemolymph from nymphs of *D. citri*. However, in the present study, *T. radiata* was not attracted by volatiles produced by 1st and 2nd instar nymphs, but was attracted by volatiles emitted from 3rd, 4th, and 5th instar nymphs. This phenomenon may be related to the vison, tactile sense, and concentration threshold of *D. citri* nymphs for *T. radiata*, individually or in combination.

This study investigated the relationship between olfactory and ovipositional preference of *T. radiata* in order to better understand the role semiochemicals play in host recognition by the parasitoid. The results indicate that there are not only olfactory, but also other factors involved in host recognition. These factors would be likely to affiliate with contact chemical stimuli, texture, colour, movement, sound, or other elements, because parasitoid reproduction is related to both a physiological and ecological context, such as nutrient allocation, utilization, and acquisition [17,38,39,40]. In further studies, those factors should be checked for recognition activity in order to aid in the understanding of host-finding behavior. In addition, in our present study, we found that female *T. radiata* presented a higher parasitism rate on 5th instar nymphs compared with those on 4th and 3rd instar nymphs. This phenomenon might be related to larval nutritional conditions and body size; favorable larval nutritional conditions give rise to increased progeny size. Additionally, a larger body size may also offer more resource for host locating [40].

The Y-tube olfactometer assays carried out with *T. radiata* males demonstrated that male parasitoids did not show attraction to any volatiles from the host insect themselves. However, *T. radiata* males have exhibited attraction to volatiles of the female *T. radiata* conspecifics compared with blank controls [8]. These investigations indicated that *T. radiata* males may forage for females in response to a volatile pheromone produced by the female. Similar findings have been observed with other parasitoid species [36].

On the basis of the results of this study, volatiles are important in the selection of hosts by parasitoids. The preference of female *T. radiata* for *M. paniculata* might be of benefit to the survival and development of *M. paniculata* and *T. radiata*. This phenomenon might be related to mutual evolution between the host plant and the parasitoids. Plants always have interactions with parasitoids that consume from the herbivores [41]. Specifically, for parasitoids that inhabit plants, certain plant characteristics can affect parasitoid behavior and associated parasitoid–herbivore interactions. Therefore, there is no doubt that plants may use some mechanisms to protect themselves for surviving in nature. For example, plants can enhance the effectiveness of herbivores’ enemies and employ such enemies as ‘bodyguards’ [42]. Specifically, parasitoids use synomones such as volatiles from the plant directly or indirectly via herbivore-induced mechanisms to locate their habitat (long-range searching). Following this, volatiles that are emitted from the host insect or its by-products are used as kairomones in the process of host location (short-range searching) [17,28,29]. It is thus possible that *T. radiata* firstly utilize volatiles that are emitted from *M. paniculata* to locate their host plant, then use volatiles produced by nymphs of *D. citri* to locate their host insect, and then undertake their feeding or parasitism.

Research on location of hosts and host habitat are important for biological control. There is a demand to identify the specific semiochemicals related with the process of host location [43,44,45]. Incorporating knowledge of the olfactory cues that parasitoid wasps respond to with the results of studies to test host specificity will afford a better understanding of all steps resulting in successful parasitism in the context of biological control. In the present study, the results indicated that *T. radiata* individuals responded to chemical cues specific to *M. paniculata* and *D. citri* 3rd, 4th, and 5th instar nymphs. These results indicate that there are not only olfactory, but also other factors involved in host location. These results can be used to guide further investigations on the searching behaviour of this parasitoid species.

## 5. Conclusions

In the present study, a Y-tube olfactometer was employed to investigate whether the parasitoid *T. radiata* is capable of utilizing the odour source that is emitted by both plants and insect hosts during its foraging. The results indicated that male *T. radiata* did not respond positively to *D. citri* instar nymphs, whereas female *T. radiata* showed a clear preference to *M. paniculata* and 3rd, 4th, and 5th instar nymphs of *D. citri*, but could not distinguish between them using olfactory cues only. In addition, nymph-stage choice for parasitism determined that *T. radiata* female adults prefer to choose late-instar nymphs for parasitism when different nymphal stages of *D. citri* are present.

## Figures and Tables

**Table 1:** The parasitism of female *Tamarixia radiata* when offered the same ratio of different *Diaphorina citri* instars nymphs for 24 h under laboratory conditions.

Host Stage Combination	Host Stage	Parasitism (%)
5th vs. 3rd instars	5th instar	77.90 ± 13.26 a
	3rd instar	8.91 ± 6.23 b
5th vs. 4th instars	5th instar	83.90 ± 5.85 a
	4th instar	26.10 ± 8.01 b
4th vs. 3rd instars	4th instar	63.20 ± 8.54 a
	3rd instar	3.05 ± 1.25 b
5th vs. 4th vs. 3rd instars	5th instar	49.57 ± 11.31 a
	4th instar	11.10 ± 1.19 b
	3rd instar	0.10 ± 0.00 b

Means followed by the different letter within different column are significantly different (Student’s *t*-test, LSD, *P* < 0.05).
