# Correction: Liu et al. *Tamarixia radiata* Behaviour Is Influenced by Volatiles from Both Plants and *Diaphorina citri* Nymphs. *Insects* 2019, *10*, 141

**DOI:** 10.3390/insects15080614

**Published:** 2024-08-15

**Authors:** Yan-Mei Liu, Shu-Hao Guo, Fei-Feng Wang, Li-He Zhang, Chang-Fei Guo, Andrew G. S. Cuthbertson, Bao-Li Qiu, Wen Sang

**Affiliations:** 1 Key Laboratory of Bio-Pesticide Innovation and Application, Department of Entomology, South China Agricultural University, Guangzhou 510640, China; yml759473267@163.com (Y.-M.L.); guoshuhaozd@163.com (S.-H.G.); wff1013634299@163.com (F.-F.W.); baileyqiu@scau.edu.cn (B.-L.Q.); 2 Engineering Research Center of Biological Control, Ministry of Education, Guangzhou 510640, China; shengfang447@126.com (L.-H.Z.); changfeiguo@163.com (C.-F.G.); 3 Independent Science Advisor, York YO41 1LZ, UK; andrew_cuthbertson@live.co.uk

Error in Figure/Table

In the original publication [1], there was a mistake in Figure 2 as published. Figure 2 and Figure 1 are repeated. The corrected Figure 2 appears below. The authors state that the scientific conclusions are unaffected. This correction was approved by the Academic Editor. The original publication has also been updated.

## Figures and Tables

**Figure 2:**
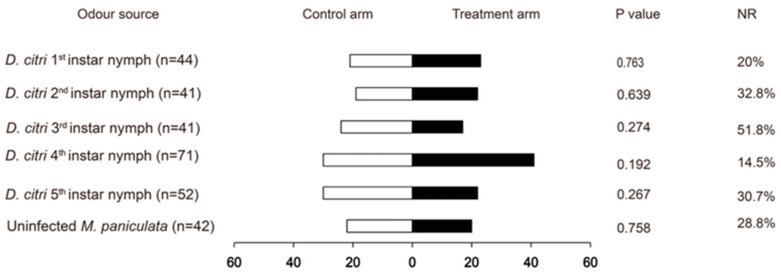
Response of male *Tamarixia radiata* to odours emitted from *Diaphorina citri* nymphs versus blank controls with a Y-tube olfactometer. NR: percentage of non-responders.
